# A Systematic Study of the Hepatic–Intestinal First-Pass Effect and Excretion Pathways of Punicalagin Based on UPLC-MS/MS

**DOI:** 10.3390/molecules31030393

**Published:** 2026-01-23

**Authors:** Zixin Chen, Zhanying Chang, Pengxia Yao, Xiaoli Gao

**Affiliations:** 1 College of Pharmacy, Xinjiang Medical University, Urumqi 830017, China; chenzixin20000510@163.com (Z.C.); changzhanying1983@163.com (Z.C.); yaopengxia_1128@163.com (P.Y.); 2 Engineering Research Center of Xinjiang and Central Asian Medicine Resources, Ministry of Education, Urumqi 830011, China

**Keywords:** punicalagin, first-pass effect, pharmacokinetics, UPLC-MS/MS, excretion, intestinal metabolism, bioavailability

## Abstract

Punicalagin, the major polyphenol in pomegranate peel, shows broad bioactivity but suffers from poor oral bioavailability. Whether hepatic or intestinal first-pass processes dominate this limitation remains unresolved. We developed a quantitative UPLC-MS/MS workflow to dissect punicalagin’s first-pass disposition and elimination in rats. Sprague–Dawley rats received punicalagin by intravenous, portal vein, oral, or intraduodenal dosing; plasma exposure was quantified by UPLC-MS/MS and analyzed noncompartmentally. We also profiled urinary and fecal excretion of punicalagin and key metabolites (punicalin, ellagic acid, urolithin C and urolithin A) to define biotransformation and clearance. Punicalagin displayed an absolute oral bioavailability of ~3.49%. First-pass analysis revealed modest hepatic extraction (~13.94%) but near-complete intestinal extraction (95.95%), identifying intestinal first-pass metabolism as the dominant barrier to systemic exposure. Consistently, parent and metabolites were eliminated mainly in feces, whereas urine contained only trace conjugated urolithin A. Collectively, these findings demonstrate that the poor oral bioavailability of punicalagin is driven primarily by extensive intestinal first-pass metabolism rather than hepatic clearance, and that its feces-dominant elimination is compatible with widespread hydrolysis and microbiota-mediated conversion within the gut. This work provides a pharmacokinetic framework to guide strategies aimed at improving oral delivery and systemic exposure of punicalagin.

## 1. Introduction

Pomegranate (*Punica granatum* L.) peel is the major by-product of pomegranate processing, accounting for approximately 30% of the fresh fruit weight [1]. Notably, it contains substantially higher levels of polyphenolic constituents than the edible arils. The peel is particularly enriched in ellagitannins (typified by punicalagin), along with ellagic acid and gallic acid, and also comprises a diverse array of flavonoids, anthocyanins, and organic acids [2,3]. Consequently, pomegranate peel is undergoing a conceptual shift from an “agricultural waste” to a strategically important, sustainable reservoir of natural polyphenols [4]. A growing body of evidence indicates that pomegranate peel polyphenols exert pronounced antioxidant, anti-inflammatory, antimicrobial, antitumor, and cardioprotective activities [5,6,7]. Mechanistically, these effects have been attributed to free-radical scavenging, metal-ion chelation, and modulation of key signalling cascades, including the NF-κB and MAPK pathways [5,8]. Moreover, following biotransformation by the gut microbiota, pomegranate-derived ellagitannins can yield urolithins and related metabolites [9,10]. These metabolites are increasingly recognised as major bioactive mediators. Collectively, these properties support the translational potential of pomegranate peel polyphenols in the prevention and management of metabolic syndrome, cardiovascular disorders, diabetes, and multiple malignancies [6,11,12,13]. In parallel, advances in green and high-efficiency extraction platforms—such as pressurised liquid extraction, ultrasound- and microwave-assisted extraction, supercritical fluid extraction, and enzyme-assisted extraction—together with their integration with approaches including solid-state fermentation, have further improved both yield and bioactivity of pomegranate peel polyphenols. These technological developments provide a robust foundation for their downstream valorisation in functional foods, dietary supplements, and bioinspired biomedical materials.

Punicalagin is the predominant water-soluble ellagitannin in pomegranate peel and exhibits a broad spectrum of biological activities, including antioxidant, anti-inflammatory, antiviral, antimicrobial, antitumour, and anti-atherosclerotic effects [14,15,16,17,18]. However, punicalagin is a high-molecular-weight (1084.7 Da), highly polar polyphenol bearing multiple hydroxyl groups [19,20,21]—physicochemical features widely considered to impede intestinal permeation and to underpin its poor oral bioavailability. Early studies provided direct evidence of limited systemic exposure. In a long-term feeding study in Sprague–Dawley rats receiving a diet containing 6% punicalagin, Cerdá and colleagues [22] reported extremely low circulating levels. Subsequent clinical investigations yielded concordant observations. In a randomized crossover trial in healthy volunteers administered pomegranate extracts with different compositions, González-Sarrías et al. [23] detected plasma concentration–time profiles only for ellagic acid, with a C_max_ range of 12–360 nM (PE-1: 74.8 ± 54.4 nM; PE-2: 64.1 ± 76.8 nM). In a separate single-dose study using a standardized pomegranate extract [(Pomella^®^ was purchased from Verdure Sciences, Inc. (Noblesville, IN, USA; Verdure Sciences)] in healthy participants, Wang and colleagues [24] reported pharmacokinetic parameters for ellagic acid and conjugated urolithin A; following enzymatic deconjugation with β-glucuronidase and sulfatase, the C_max_ values for ellagic acid versus urolithin A conjugates were (35.3 ± 17.8 vs. 77.8 ± 28.1) ng·mL^−1^, and the corresponding AUC_0–24h_ values were (848.5 ± 427.9 vs. 1738 ± 584.9) h·ng·mL^−1^. More recently, Chang et al. [25] estimated the absolute oral bioavailability of punicalagin in rats to be only ~3.22–5.38%. With respect to biotransformation, punicalagin is readily hydrolysed in the small intestine to yield ellagic acid and is further converted by the gut microbiota into urolithins and related dibenzopyranone derivatives [26]. Consistent with this pathway, Cerdá et al. [22] detected ellagic acid, urolithins A, B, and C, and their corresponding phase II metabolites in plasma, faeces, and urine; later work suggested that urolithin A, urolithin B, and isourolithin A constitute the major terminal metabolites [23,27]. Despite these advances, the global pharmacokinetic fate of punicalagin—and, critically, the rate-limiting steps that drive its low oral bioavailability—remains insufficiently defined owing to a lack of systematic and direct in vivo evidence. Available findings suggest that this phenomenon likely results from the combined action of multiple. As a structurally complex polyphenolic compound, punicalagin undergoes progressive breakdown in the gastrointestinal tract and is further converted by intestinal microorganisms into downstream derivatives, leading to a substantial shift in the predominant forms that ultimately reach the systemic circulation. After absorption through the intestinal epithelium and subsequent entry into the liver, these products typically undergo rapid conjugation reactions and circulate mainly in conjugated forms before being eliminated predominantly via urine [28,29]. Clinical observations have shown that related metabolites can be detected in urine for extended periods, further supporting a continuous sequence of intestinal transformation, absorption, conjugation, and excretion [30]. Meanwhile, long-term animal studies indicate that only a small proportion of ingested punicalagin can be recovered as identifiable products in urine and feces, suggesting that intestinal conversion, early processing in the body, and diversion into elimination pathways together determine its overall distribution and excretion profile [22]. Three fundamental questions, in particular, remain unresolved: (1) during oral absorption, how can the respective contributions of intestinal and hepatic first-pass elimination of punicalagin be quantitatively disentangled? (2) what are the dominant elimination routes for punicalagin and its major metabolites, and what kinetic features characterize their excretion? and (3) is there an intrinsic linkage between the magnitude of first-pass metabolism and excretory behaviour? Addressing these questions in a rigorous and integrated manner is essential for elucidating exposure mechanisms of punicalagin in vivo, evaluating its feasibility as a drug candidate or functional ingredient, anticipating potential drug–drug and drug–food interactions, and rationally guiding the design of next-generation delivery systems.

## 2. Results and Discussion

### 2.1. Method Validation

#### 2.1.1. Specificity

**Figure 1:**
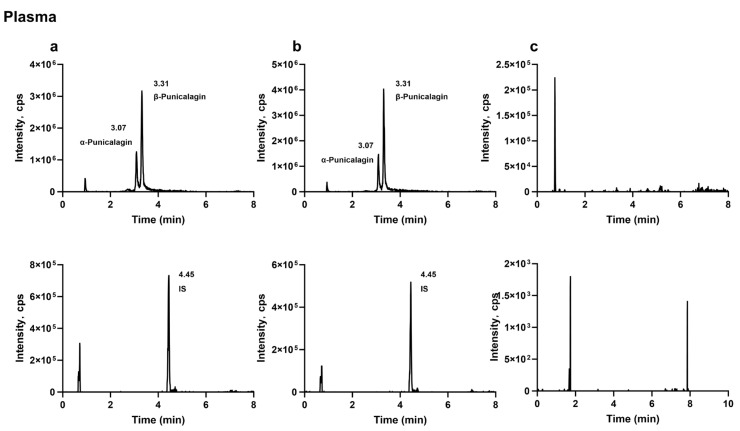
Typical extracted-ion MRM chromatograms of punicalagin and the internal standard in plasma: (**a**) plasma sample collected 2 h after oral administration of punicalagin (200 mg·kg^−1^) with internal standard; (**b**) blank plasma spiked with punicalagin and internal standard; (**c**) blank plasma.

**Figure 2:**
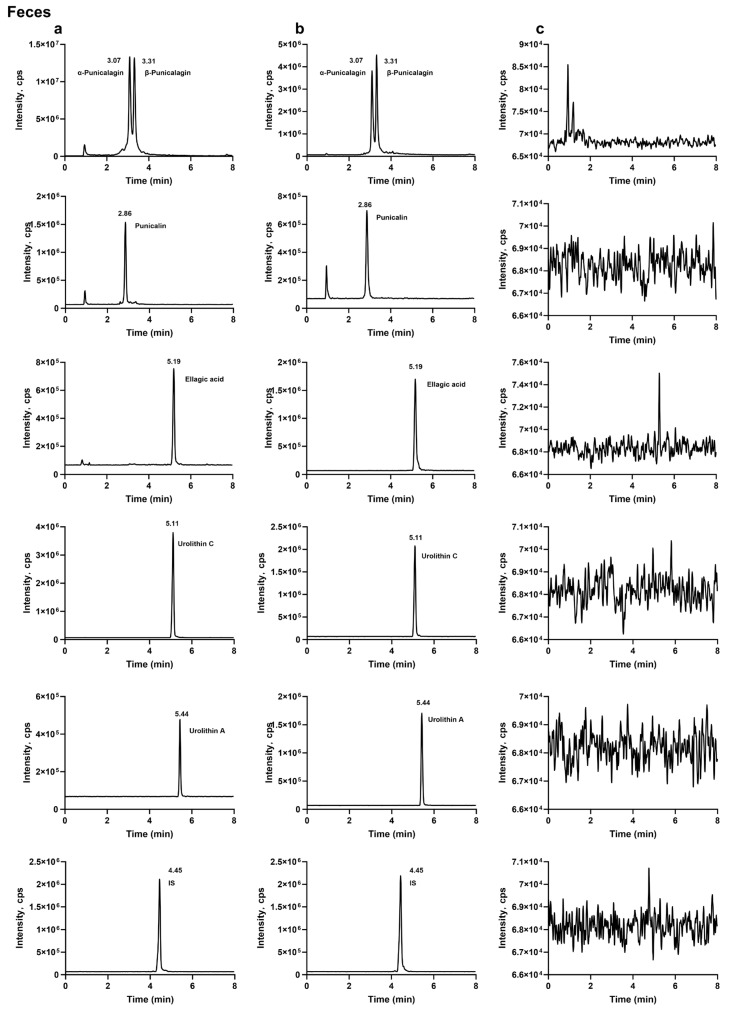
Typical extracted-ion MRM chromatograms of punicalagin, punicalin, ellagic acid, urolithin C, urolithin A, and the internal standard in feces: (**a**) fecal sample collected 2 h after oral administration of punicalagin (200 mg·kg^−1^) with internal standard; (**b**) blank feces spiked with punicalagin, punicalin, ellagic acid, urolithin C, urolithin A, and internal standard; (**c**) blank feces.

**Figure 3:**
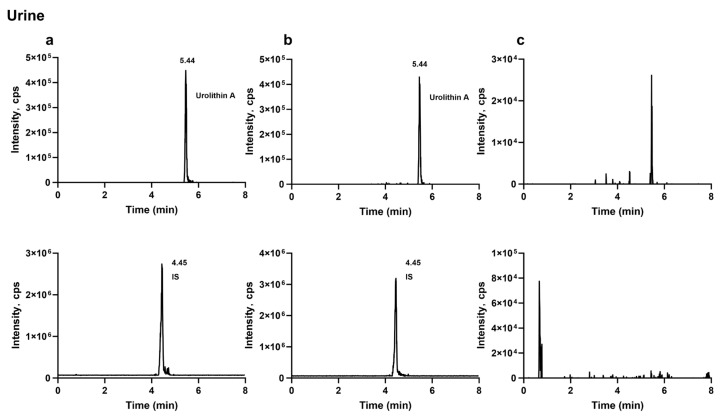
Typical extracted-ion MRM chromatograms of urolithin A and the internal standard in urine: (**a**) urine sample collected 24 h after oral administration of punicalagin (200 mg·kg^−1^) with internal standard; (**b**) blank urine spiked with urolithin A; (**c**) blank urine.

The analytes and the internal standard were well separated, and no apparent endogenous interference or cross-interference was observed at their retention times. Representative MRM chromatograms of blank biological samples, blank samples spiked with the analytes and the internal standard, and biological samples collected after oral administration of the analytes are shown in Figure 1, Figure 2 and Figure 3.

#### 2.1.2. Linearity

The calibration curves, correlation coefficients, and linear ranges for each analyte in plasma, urine, and feces are summarized in Table 1. All correlation coefficients (R) were greater than 0.99, indicating good linearity. The lower limit of quantification for punicalagin in plasma was 1.0 μg·mL^−1^. In feces, the lower limits of quantification for punicalagin, punicalin, ellagic acid, urolithin C, and urolithin A were 1.0, 0.1, 0.1875, 0.03125, and 0.0625 μg·mL^−1^, respectively. The lower limit of quantification for urolithin A in urine was 0.05 μg·mL^−1^.

#### 2.1.3. Precision and Accuracy

Precision and accuracy were evaluated in plasma, urine, and feces using quality control samples at three concentration levels (Table 2). The within-run and between-run relative standard deviations ranged from 3.25% to 14.76% and from 1.04% to 10.57%, respectively, while the relative errors ranged from −10.71% to 11.59%. These results indicate that the method performance was within acceptable limits and met the requirements for bioanalytical evaluation.

#### 2.1.4. Extraction Recovery and Matrix Effects

Extraction recovery and matrix effects for the analytes are summarized in Table 3. The mean extraction recoveries in plasma, urine, and feces ranged from 87.27% to 111.60%, with no significant differences across the three quality control levels. Matrix effects for all analytes in the different matrices were within acceptable limits, ranging from 85.49% to 113.64%. These results indicate that no obvious signal enhancement or suppression was observed in rat plasma, feces, or urine under the validated conditions, supporting the suitability of the method for determining the analytes in these matrices.

#### 2.1.5. Stability

Stability results are summarized in Table 4. All analytes remained stable under the tested conditions: after 24 h in the autosampler (4 °C), the extracted plasma, urine, and fecal samples showed relative errors of −12.92% to 11.31% and relative standard deviations of <13.48%; after three freeze–thaw cycles, relative errors were −12.74% to 12.41% with relative standard deviations of <12.44%; and after long-term storage at −20 °C for 14 days, relative errors were −13.97% to 13.43% with relative standard deviations of <12.75%. These results indicate that the samples exhibited good stability under the above storage and handling conditions.

### 2.2. Assessment of Hepatic and Intestinal Availabilities in Rats

**Figure 4:**
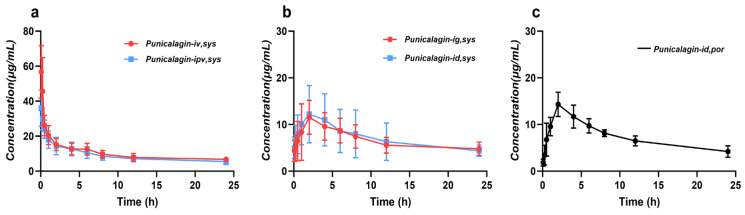
Plasma concentration–time profiles of Punicalagin different routes of administration. (**a**), Mean plasma concentration–time curves after intravenous and portal-vein injection at 10 mg·kg^−1^. (**b**), Mean systemic (peripheral) plasma concentration–time curves after oral gavage and duodenal administration at 200 mg·kg^−1^. (**c**), Mean portal-vein plasma concentration–time curve after duodenal administration at 200 mg·kg^−1^ (Mean ± SD, *n* = 6).

Under different administration routes (oral, intravenous, portal vein, and intraduodenal), the mean concentration–time profiles of punicalagin in the portal vein or ophthalmic venous plexus are shown in Figure 4, with key pharmacokinetic parameters summarized in Table 5 and Table 6. Comparison across administration routes provides direct in vivo evidence for the primary factors limiting systemic exposure of punicalagin. Overall, systemic exposure following portal vein and intravenous administration was comparable, indicating that hepatic first-pass clearance of the parent compound is not the dominant factor. In contrast, under oral or intraduodenal administration, despite minimal loss in the gastric phase, the fraction reaching systemic circulation remained extremely low, suggesting that the intestinal phase constitutes the main bottleneck limiting oral bioavailability. Therefore, the low oral bioavailability of punicalagin can be interpreted as primarily resulting from restricted parent compound exposure during the intestinal phase, rather than the conventional assumption of liver-dominated first-pass metabolism. Mechanistically, intestinal loss likely arises from multiple synergistic processes, with a substantial portion occurring prior to or during absorption. Punicalagin is a high-molecular-weight polyphenolic tannin, chemically predisposed to depolymerization and hydrolysis. Previous studies and in vivo metabolic patterns indicate that punicalagin undergoes depolymerization and hydrolysis in the gastric–small intestinal environment to generate ellagic acid, which is subsequently transformed by colonic microbiota into smaller metabolites such as urolithins, naturally limiting systemic exposure of the parent compound. This mechanism explains why, under oral administration, even minimal hepatic first-pass clearance does not result in significant systemic exposure of the parent molecule. Furthermore, even when some parent compound reaches the small intestinal epithelium, its transmembrane absorption may be restricted by physicochemical properties. Punicalagin’s high molecular weight, high polarity, and multiple hydrogen-bond donor and acceptor sites hinder passive diffusion, while chemical and enzymatic transformation in the intestinal lumen and epithelium, microbial metabolism, hydrolysis promoted by the digestive environment, and efflux transporters (e.g., P-glycoprotein) may further limit absorption [31,32,33]. Compared with the intestinal phase, hepatic first-pass metabolism of the parent compound is relatively limited, although the liver remains important for the overall in vivo fate. For absorbed small molecules such as ellagic acid, urolithins, and their derivatives, phase II metabolism in the liver and intestinal epithelium (UGT/SULT-mediated glucuronidation and sulfation) is more pronounced, resulting in circulating and urinary forms predominantly as conjugates, representing key features of systemic exposure and excretion [32,33].

It should be noted that when absolute oral bioavailability is very low, terminal plasma concentrations in the oral group may fall below the lower limit of quantification (LLOQ) prematurely, leading to insufficient data points for terminal-phase fitting. This may result in underestimation of AUC (especially AUC_t–∞_) in non-compartmental analysis, and consequently, underestimation of oral bioavailability. To minimize this methodological bias, subsequent studies should extend the sampling duration and ensure that at least consecutive quantifiable points are obtained at the terminal phase, thereby improving the robustness of terminal-phase parameter estimation.

In summary, this study using different administration routes indicates that both hepatic and intestinal first-pass processes reduce systemic exposure, with intestinal loss being predominant and likely representing the key limitation to oral bioavailability of punicalagin. This mechanistic framework aligns with previously reported low oral bioavailability [25] and provides a clear direction for future investigations focused on digestive stability and transformation, epithelial permeability, efflux transporter contribution, and metabolite conjugate profiling.

### 2.3. Excretion Study

A substantial body of work has addressed the excretion kinetics of punicalagin. Using rats, Cerdá et al. [22] administered a 6% punicalagin diet for 37 days and continuously collected urine and feces for HPLC-MS/MS analysis, reporting that identifiable metabolites recovered in feces and urine collectively accounted for ~3–6% of the ingested amount. In a complementary human study, five healthy volunteers consumed 1 L of pomegranate juice daily for five consecutive days (total polyphenols 5.58 g/L, including 4.37 g/L punicalagin). Neither punicalagin nor ellagic acid was detected in plasma or urine; only three ellagic acid-derived metabolites were observed, and the total urinary excretion of metabolites corresponded to 0.7–52.7% of the ingested punicalagin dose [34]. Shi et al. provided rats with pomegranate peel polyphenol extract for three weeks (300 mg/kg/day) and quantified only cumulative urinary excretion of urolithin A (~1.71 mg) [35]. Collectively, these studies predominantly reflect long-term interventions and typically quantify only one or a limited subset of constituents. This study provides the first systematic investigation of the excretion of punicalagin and its four metabolites.

**Figure 5:**
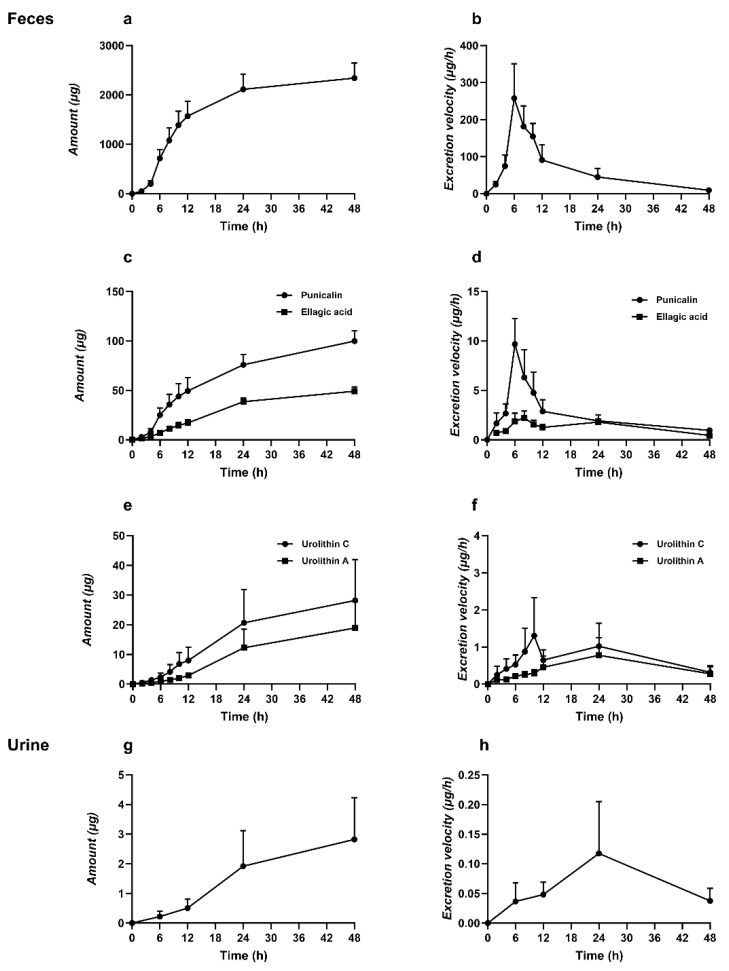
After oral administration of punicalagin (200 mg·kg^−1^) in rats, (**a**,**b**) represent the fecal excretion amount and excretion rate of punicalagin; (**c**,**d**) represent the fecal excretion amount and excretion rate of punicalin and ellagic acid; (**e**,**f**) represent the fecal excretion amount and excretion rate of urolithin A and C; and (**g**,**h**) represent the urinary excretion amount and excretion rate of conjugated urolithin A (Mean ± SD, *n* = 6).

**Figure 6:**
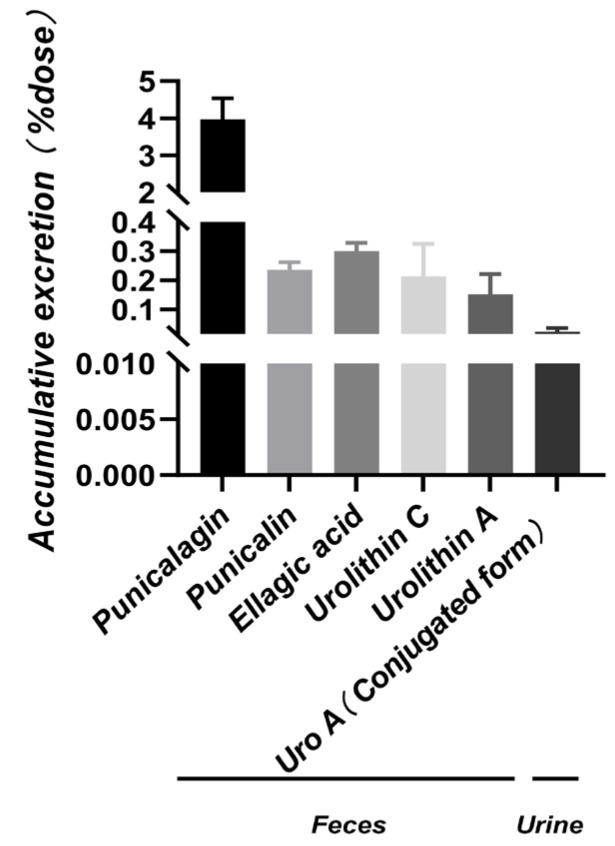
Cumulative fecal excretion percentages of punicalagin, punicalin, ellagic acid, urolithin C, and urolithin A, and cumulative urinary excretion percentage of conjugated urolithin A, in rats following oral administration of punicalagin (200 mg·kg^−1^) (Mean ± SD, *n* = 6).

After oral administration of punicalagin in rats, the cumulative excretion of each compound in feces and urine is shown in Figure 5 and Figure 6. A UPLC-MS/MS method was developed to quantitatively measure punicalagin and four unconjugated metabolites. Following a single oral dose of 200 mg·kg^−1^, the cumulative fecal excretion of punicalagin was 2340.93 ± 304.52 μg (Figure 5a), with a peak excretion rate at 6 h post-dose (257.87 ± 92.92 μg/h, Figure 5b). Feces accounted for the primary elimination route over 48 h (~4.88% of the dose, Figure 6). This early-phase rapid clearance aligns with the high molecular weight, polarity, and limited membrane permeability characteristic of tannins, including punicalagin, suggesting that a substantial portion is rapidly excreted as unabsorbed parent compound or after limited gastrointestinal degradation. Previous studies have indicated that tannins undergo hydrolysis in the gastrointestinal tract to release ellagic acid (EA), which is subsequently metabolized by colonic microbiota into urolithins, resulting in a delayed appearance of downstream metabolites [10,33]. Additionally, punicalagin undergoes ester hydrolysis and tannin cleavage in the gastrointestinal tract, generating lower-molecular-weight polyphenols, including punicalin and ellagic acid. Their cumulative fecal excretion was 99.90 ± 10.60 μg and 49.13 ± 4.42 μg, respectively (Figure 5c), with peak excretion rates at 6 h and 8 h post-dose (8.62 ± 3.74 μg/h and 2.23 ± 0.68 μg/h, Figure 5d), indicating early peak excretion. As ellagic acid serves as the direct precursor for urolithin formation, downstream metabolites appear with a time delay [10,33]. Under colonic microbiota action, cumulative fecal excretion of urolithin C and urolithin A was 27.93 ± 14.05 μg and 18.94 ± 8.90 μg, respectively (Figure 5e), with peak excretion rates at 10 h and 24 h post-dose (1.31 ± 1.03 μg/h and 0.78 ± 0.47 μg/h, Figure 5f), consistent with delayed production governed by microbial metabolism and colonic residence time [36,37]. Punicalagin, punicalin, ellagic acid, and urolithin C were not detected in urine, likely due to the high molecular weight and polarity of punicalagin and punicalin, which limits glomerular filtration and favors fecal elimination [38]. Ellagic acid and urolithin C may appear in urine mainly as polar conjugates. In our study, no conjugates were detected, possibly due to limited sampling duration. Only urolithin A was observed, with a cumulative urinary excretion of 2.73 ± 1.31 μg (Figure 5g) and a peak excretion rate at 24 h post-dose (0.12 ± 0.09 μg/h, Figure 5h), further indicating that absorbed urolithins are primarily excreted in urine as phase II conjugates (glucuronides or sulfates) [23,30]. Furthermore, phase II conjugation increases molecular weight and hydrophilicity; compounds with molecular weight ≥400–500 Da and multiple anionic groups may also undergo biliary excretion in addition to renal clearance [38,39].

It should be noted that fecal samples were not subjected to enzymatic hydrolysis; thus, the presence of conjugates in feces could not be confirmed. However, previous evidence indicates that phase II conjugation mainly occurs post-absorption (intestinal epithelium or liver), so circulating metabolites are predominantly conjugated and typically detected in plasma or urine. Additionally, fecal enzymes, particularly β-glucuronidases, can hydrolyze conjugates back to free forms, which explains the predominance of free compounds in feces. Without enzymatic treatment, the presence of conjugates may be underestimated [40,41].

Current evidence broadly supports a tight linkage between the magnitude of intestinal first-pass effects and excretion kinetics for high-molecular-weight polyphenols (e.g., condensed tannins/proanthocyanidins and ellagitannins). On one hand, their large molecular size and high polarity favor depolymerization and/or hydrolysis within the intestinal lumen; the resulting products undergo extensive presystemic phase II metabolism in the intestinal epithelium (via UGTs and SULTs) and are further conjugated in the liver, such that the parent compounds scarcely enter the systemic circulation [42]. This coupled gut–liver first-pass process provides a direct mechanistic explanation for their characteristically low oral bioavailability and extremely low plasma exposure. On the other hand, because most of these polyphenols are poorly absorbed, they are gradually degraded by the gut microbiota into smaller phenolic acids or urolithins. After absorption, these metabolites are eliminated over prolonged periods in urine and feces largely as glucuronide and/or sulfate conjugates, whereas the unutilized high-molecular-weight polyphenols themselves are excreted predominantly in feces [43,44]. Animal and human kinetic studies further indicate that large proanthocyanidins are rarely detected as intact molecules in plasma or urine; instead, their disposition is dominated by delayed, blunted, and long-lasting metabolite excretion profiles. In some settings, the cumulative urinary recovery of metabolites can approach the ingested amount, while substantial quantities of unabsorbed polymers remain detectable in feces—together reflecting sustained intestinal first-pass processing coupled with microbial catabolism and underpinning a slow-release, long-range excretion phenotype [45,46]. Consistent with this paradigm, our data suggest that the ellagitannin punicalagin likewise exhibits poor absorption of both the parent compound and ellagic acid, but is converted in the colon to urolithins, which can persist in vivo for several days and are progressively eliminated via urine and feces. Importantly, urolithin formation is closely tied to inter-individual differences in microbial metabolic capacity, yielding marked variability in excretion trajectories across subjects [47,48]. Overall, punicalagin undergoes substantial intestinal first-pass processing and a “retention in the gut followed by microbiota-driven remodeling” sequence, resulting in a kinetic signature characterized by brief and minimal systemic exposure to the parent compound and delayed metabolite appearance and elimination that track with both first-pass intensity and gut microbial composition.

## 3. Materials and Methods

### 3.1. Reagents and Chemicals

**Figure 7:**
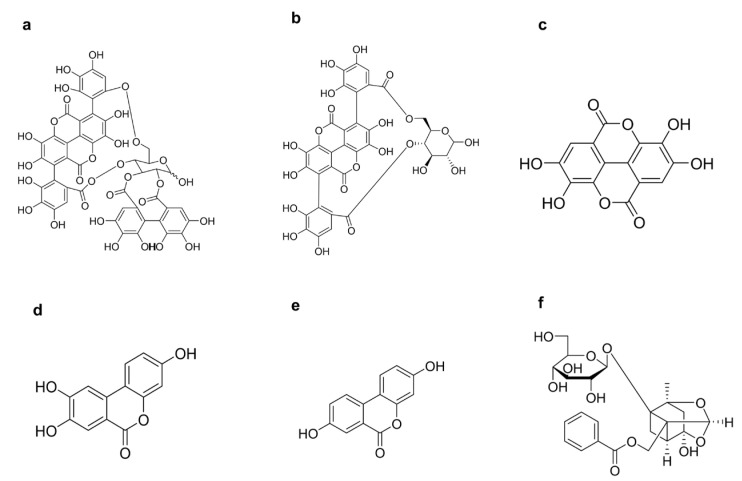
Chemical structures of (**a**) punicalagin, (**b**) punicalin, (**c**) ellagic acid, (**d**) urolithin C, (**e**) urolithin A, and (**f**) paeoniflorin (internal standard).

Punicalagin (purity 98.2%) was purchased from Chengdu Must Bio-Technology Co., Ltd. (Chengdu, China). Paeoniflorin (internal standard), punicalin, ellagic acid, urolithin C, and urolithin A were obtained from Sichuan Vicqi Bio-Technology Co., Ltd., Chengdu, China (Figure 7). Acetonitrile and methanol (LC grade) were purchased from Sigma (St. Louis, MO, USA). Purified water (Watsons) was supplied by Guangzhou Watsons Food & Beverage Co., Ltd. (Guangzhou, China). All other reagents were of commercially available analytical grade.

### 3.2. Animals

Male adult SD rats (200 ± 20 g) were purchased from the Animal Experiment Center of Xinjiang Medical University. All rats were housed in a 12/12-h light/dark cycle environment (temperature: 23 ± 1 °C, relative humidity: 60 ± 10%). Except for 12 h prior to the experiments, the animals were allowed free access to water and standard food throughout the study. All experimental procedures were performed in accordance with protocols approved by the Animal Ethics Committee of Xinjiang Medical University.

### 3.3. Preparation of Standard Solutions and Quality Control (QC) Samples

Accurately weighed reference standards of punicalagin, punicalin, ellagic acid, urolithin A, and urolithin C were dissolved in methanol to prepare stock solutions at concentrations of 10.00, 1.00, 1.00, 1.00, and 1.00 mg·mL^−1^, respectively. The corresponding stock solutions were serially diluted with methanol to generate single-analyte working solutions at multiple concentration levels for plasma and urine. For fecal samples, the individual stock solutions were mixed at predetermined ratios and then serially diluted with methanol to obtain mixed working solutions at different concentrations, which were used to construct calibration curves and prepare quality-control (QC) samples. Paeoniflorin was accurately weighed and diluted with methanol to prepare an internal-standard stock solution (1 mg·mL^−1^), and was further diluted with methanol to 10 μg·mL^−1^ immediately before use. All solutions were stored at 4 °C and equilibrated to room temperature prior to analysis.

To prepare calibration samples, 100 μL of the working solution was mixed with 900 μL of blank plasma, urine, or fecal homogenate. The concentration ranges of the calibration samples are shown in Table 1. QC samples were prepared independently using the same procedure, and their concentrations are listed in Table 7.

### 3.4. Preparation of Plasma, Feces and Urine Samples

Plasma samples were collected and processed directly without additional pretreatment. Fecal samples were air-dried at room temperature, accurately weighed, and ground to a fine powder. Methanol containing 2% formic acid was added at 20 mL/g, followed by ultrasonic extraction for 30 min in an ice bath. For urine, 1 mL aliquots were transferred into 10 mL centrifuge tubes, mixed with 980 μL acetate buffer (pH 5.0), and supplemented with 20 μL of a mixed enzyme solution containing β-glucuronidase and arylsulfatase, followed by overnight incubation at 37 °C. An aliquot (100 μL) of each biological matrix was accurately transferred into a 1.5 mL microcentrifuge tube. Internal-standard working solution (100 μL, 2 μg·mL^−1^) was added and vortex-mixed for 1 min. Protein precipitation was then performed by adding 250 μL methanol containing 2% formic acid, followed by vortex-mixing for 3 min. Samples were centrifuged at 4 °C and 14,000 rpm for 15 min. A 200 μL aliquot of the supernatant was collected, and 5 μL was injected for UPLC-MS/MS analysis.

### 3.5. UPLC-MS/MS System and Instrumental Conditions

The analytical system comprised a Waters ACQUITY UPLC coupled to a Waters Xevo TQ-S tandem mass spectrometer with an electrospray source (Waters, Milford, MA, USA). Separation was achieved on an ACQUITY UPLC^®^ HSS T3 column (1.8 μm, 2.1 mm × 100 mm). The mobile phases were 0.1% formic acid in water (A) and methanol (B), delivered at 0.3 mL/min with the following gradient: 0–1.0 min, 5% B; 1.0–4.0 min, 5–60% B; 4.0–5.5 min, 60–95% B; 5.5–6.5 min, 95% B; 6.5–7.0 min, 95–5% B; and 7.0–8.0 min, 5% B (column re-equilibration). The injection volume was 5 μL; the autosampler and column were maintained at 4 °C and 30 °C, respectively.

Quantification was performed in negative-ion mode using targeted MS/MS monitoring (multiple reaction monitoring). After source optimization, the instrument was operated with the following settings: source temperature 150 °C; desolvation temperature 400 °C; cone voltage 90 V; capillary voltage 2.5 kV; desolvation gas flow 800 L/h; cone gas flow 150 L/h; and nebulizer gas pressure 7 bar. Because the monitored ion transitions and related voltage/energy settings varied across compounds, these parameters were optimized for each analyte (Table 8). Data acquisition, instrument control, and processing were performed using MassLynx 4.1 (Waters, Milford, MA, USA).

### 3.6. Method Validation

The optimized UPLC-MS/MS method was validated in accordance with the US Food and Drug Administration (FDA) guidance on bioanalytical method validation. Validation encompassed selectivity/specificity, linearity, accuracy and precision, extraction recovery, matrix effects, and stability in plasma, urine, and feces to ensure compliance with predefined acceptance criteria [49]. validation experiments were conducted using at least six independent sources of blank matrix for each biological matrix.

#### 3.6.1. Specificity

Specificity was evaluated by comparing chromatograms of blank rat plasma, feces, and urine from six individual sources with those of corresponding blank matrices spiked at the mid-level QC concentration and with incurred samples (plasma and fecal samples collected 2 h after oral dosing and urine samples collected 24 h after dosing). The absence of significant endogenous interference was confirmed at the retention times of each analyte and the internal standard (IS).

#### 3.6.2. Lower Limit of Quantification and Calibration Curve

To assess linearity, calibration curves were constructed by plotting the peak area ratio (y) versus the analyte concentration (x) in plasma, urine, and feces. The lower limit of quantification (LOQ) was defined as the lowest concentration on the calibration curve that could be quantified reliably; at this level, the signal-to-noise ratio was required to be ≥10. Acceptance criteria were as follows: for all concentration levels except the LOQ, the deviation from nominal values had to be within ±15%, while the allowable deviation at the LOQ was within ±20%.

#### 3.6.3. Precision and Accuracy

Precision and accuracy were evaluated using quality control samples at three concentration levels. Within-run performance was assessed using six independent determinations on the same day, and between-run performance was assessed over three consecutive days. Accuracy was expressed as relative error between the mean measured value and the nominal value, and precision was expressed as relative standard deviation of the measured values. For each concentration level, the relative standard deviation was required to be ≤15%, and the relative error had to be within ±15%.

#### 3.6.4. Extraction Recovery and Matrix Effects

Extraction recovery was determined by comparing peak areas obtained from samples spiked before extraction (pre-extraction spiked) with those from samples spiked after extraction (post-extraction spiked) at equivalent nominal concentrations. Recovery was evaluated for both analytes and the IS, and repeatability was considered acceptable if the RSD was ≤15% at each QC level. Matrix effects were evaluated by comparing peak areas from post-extraction spiked samples with those from neat standard solutions at the same nominal concentrations to obtain the matrix factor (MF). To control for variability in ion suppression/enhancement, the IS-normalized MF was calculated. Matrix effects were considered acceptable if the IS-normalized MF was within 85–115% with an RSD ≤ 15% across six independent matrix sources at each QC level. Results are presented as mean ± standard deviation (SD).

#### 3.6.5. Stability

Stability of all analytes in plasma, urine, and feces was assessed using six replicate quality control samples at each of three concentration levels. Conditions included short-term storage (4 °C for 24 h), long-term storage (−20 °C for 2 weeks), and three freeze–thaw cycles. Stability was evaluated by comparing measured concentrations with nominal concentrations and calculating relative standard deviation and relative error; for each concentration level, the mean deviation was required to be within ±15%.

### 3.7. In Vivo Pharmacokinetic Study in Rats

#### 3.7.1. Surgical Cannulation in Rats

Cannulated rats were used to investigate the difference in the first-pass effect of anisodine glycoside between the liver and intestine. The surgical procedure was as follows: after an overnight fast, rats were anesthetized with 5% pentobarbital sodium (50 mg·kg^−1^, i.p). A 2-cm midline incision was made in the abdomen, and the pyloric vein was gently isolated. A heparinized polyethylene catheter was then inserted into the pyloric vein and advanced partially toward the portal vein. This catheter was used for portal vein dosing or for collection of portal blood samples.

#### 3.7.2. Whole-Body Pharmacokinetic Study

The overall bioavailability can be expressed as F_meas_ = F_a_ × F_g_ × F_h_, where F_h_ represents the fraction escaping hepatic first-pass metabolism, and F_a_·F_g_ denotes intestinal bioavailability, composed of the fraction absorbed across the gastrointestinal tract (F_a_) and the fraction that avoids intestinal metabolism (F_g_) [50,51,52].

Hepatic bioavailability was evaluated by comparing the systemic AUCs following portal vein and intravenous administration. Anisodine glycoside was dissolved in 0.9% saline. Male Sprague–Dawley rats were randomly assigned to two groups (*n* = 6 per group) and administered anisodine glycoside (10 mg·kg^−1^) via either the portal vein or tail vein. Blood samples (~300 μL) were collected from the retro-orbital venous plexus at 0.083, 0.25, 0.5, 1, 2, 4, 6, 8, 12, and 24 h post-dose. Samples were centrifuged at 4 °C and 4000 rpm for 15 min, each collected plasma sample was analyzed three times using UPLC-MS/MS.

Three additional groups of rats (*n* = 6 per group) were used to determine intestinal bioavailability. Anisodine glycoside was prepared as described above. Following oral administration or intraduodenal injection (200 mg·kg^−1^), blood samples (~300 μL) were collected from either the portal vein or retro-orbital venous plexus at 0.083, 0.25, 0.5, 1, 2, 4, 6, 8, 12, and 24 h. Plasma was obtained after centrifugation at 4 °C and 4000 rpm for 15 min, Each collected plasma sample was analyzed three times using UPLC-MS/MS. Pharmacokinetic parameters were derived by non-compartmental analysis using PKSolver 2.0. Total bioavailability (F_meas_), gastric bioavailability (F_G_), hepatic bioavailability (F_h_), intestinal bioavailability (F_a_·F_g_), and hepatic (ER_h_) and intestinal (ER_g_) extraction ratios were calculated using the following formulas:

(1) $$F\;=\;\frac{{\mathrm{AUC}}_{\mathrm{ig},\mathrm{sys}}}{{\mathrm{AUC}}_{\mathrm{iv},\mathrm{sys}}}\;\times \;\frac{{\mathrm{Dose}}_{\mathrm{iv},\mathrm{sys}}}{{\mathrm{Dose}}_{\mathrm{ig},\mathrm{sys}}}$$

(2) $$\;F_{h}=\frac{{\mathrm{AUC}}_{\mathrm{ipv},\mathrm{sys}}}{{\mathrm{AUC}}_{\mathrm{iv},\mathrm{sys}}}\;\times \;\frac{{\mathrm{Dose}}_{\mathrm{iv},\mathrm{sys}}}{{\mathrm{Dose}}_{\mathrm{ipv},\mathrm{sys}}}$$

(3) $$\;{\mathrm{ER}}_{h}=1\;-\;F_{h}\;$$

(4) $$F_{G}=\frac{{\mathrm{AUC}}_{\mathrm{id},\mathrm{sys}}}{{\mathrm{AUC}}_{\mathrm{ig},\mathrm{sys}}}\;\times \;\frac{{\mathrm{Dose}}_{\mathrm{ig},\mathrm{sys}}}{{\mathrm{Dose}}_{\mathrm{id},\mathrm{sys}}}$$

(5) $$F_{a}\;\times \;F_{g}=\frac{Q_{\mathrm{por}}\;\times \;R_{b\;}\times \;({\mathrm{AUC}}_{\mathrm{id},\mathrm{por}}\;-\;{\mathrm{AUC}}_{\mathrm{id},\mathrm{sys}})}{{\mathrm{Dose}}_{\mathrm{id}}}$$

(6) $${\mathrm{ER}}_{g}=1\;-\;F_{g}\;\times \;F_{a}$$

(7) $$F_{\mathrm{meas}}=F_{g}\;\times \;F_{a}{\;\times \;F}_{h}\;$$

Among these parameters, AUC_ig,sys_, AUC_id,sys_, AUC_iv,sys_, and AUC_ipv,sys_ represent the systemic AUCs following oral, intraduodenal, intravenous, and portal vein administration, respectively. AUCi_d,por_ denotes the portal venous AUC after intraduodenal administration. The portal blood flow rate (Q_por_) was set at 32.9 mL·min^−1^·kg^−1^ [51]. The blood-to-plasma concentration ratio (R_b_) was determined using an in vitro method [50]. Briefly, anisodine glycoside was added to freshly collected mixed rat blood at a final concentration of 25 μg·mL^−1^. After incubation at 37 °C for 15 min, a 100 μL aliquot of the blood sample was collected, and the remaining blood was centrifuged to obtain 100 μL of plasma. Both blood and plasma samples were processed for protein precipitation as described above and analyzed using UPLC-MS/MS. The R_b_ value was calculated as the ratio of the drug concentration in whole blood to that in plasma.

### 3.8. Excretion Study

For the urinary and fecal excretion study, six rats received a single oral dose of punicalagin (200 mg·kg^−1^) using the same procedure described above. After dosing, rats were housed individually in metabolic cages for the separate collection of feces and urine. Fecal samples were collected before dosing and over the following post-dose intervals: 0–2, 2–4, 4–6, 6–8, 8–10, 10–12, 12–24, and 24–48 h. Urine samples were collected over 0–6, 6–12 12–24, and 24–48 h post-dose. The dry weight of fecal samples and the urine volume were recorded. All fecal and urine samples were stored at −80 °C until analysis. Each fecal and urine sample was analyzed in triplicate using UPLC-MS/MS.

### 3.9. Data Analysis

Data are expressed as mean ± standard deviation (SD). Differences between two groups were assessed using the *t*-test, while multiple-group comparisons were performed by one-way analysis of variance (ANOVA). A *p* value of <0.05 was considered statistically significant.

## 4. Conclusions

We developed and validated a rapid and sensitive UPLC-MS/MS method for the quantitative analysis of punicalagin and related metabolites in biological samples. By comparing the in vivo disposition of punicalagin following oral, intravenous, portal vein, and intraduodenal administration, together with an analysis of excretion profiles, we evaluated the primary sites of first-pass disposition and their relationship to elimination. Under the dosing and sampling conditions of this study, intestinal processes exerted a more pronounced impact on systemic exposure, whereas the contribution of hepatic first-pass clearance was limited. In addition, loss during the gastric phase was not a key factor constraining overall exposure. After oral administration, punicalagin and several metabolites were detected predominantly in feces, while only minor signals related to urolithin A were observed in urine. Collectively, these findings suggest that punicalagin undergoes substantial transformation in the gastrointestinal tract and is further influenced by gut microbiota-mediated metabolism, thereby maintaining low systemic exposure of the parent compound and causing a time-lagged appearance and clearance of downstream metabolites. These results provide pharmacokinetic evidence to support future optimization of oral delivery, with emphasis on the intestinal barrier, intestinal metabolic processes, and gut microbiota-mediated biotransformation.

## Figures and Tables

**Table 1:** Calibration curves, correlation coefficients, and linear ranges for punicalagin, punicalin, ellagic acid, urolithin A, and urolithin C in different matrices determined during method validation using the UPLC-MS/MS method (*n* = 6).

Matrix	Analyte Compounds	Linear Range (μg·mL^−1^)	Linear Regression Equation	Correlation Coefficient (*R*)	LLOQ (μg·mL^−1^)
Plasma	Punicalagin	1.00–250	y = 0.1501x − 0.0997	0.9964	1.00
Feces	Punicalagin	1.00–200	y = 0.0501x − 0.0614	0.9994	1.00
	Punicalin	0.1875–37.5	y = 0.0969x − 0.0215	0.9999	0.1875
	Ellagic acid	0.10–20	y = 0.3519x + 0.0191	0.9997	0.1
	Urolithin C	0.03125–6.25	y = 1.1147x + 0.0625	0.9991	0.03125
	Urolithin A	0.0625–12.5	y = 0.486x + 0.0313	0.9997	0.0625
Urine	Urolithin A	0.050–40	y = 0.2362x + 0.0731	0.9996	0.05

**Table 2:** Intra-day and inter-day precision for the determination of punicalagin in rat plasma, urolithin A in urine, and punicalagin, punicalin, ellagic acid, urolithin C, and urolithin A in feces using the UPLC-MS/MS method (*n* = 6; three or four concentration levels: LLOQ, LQC, MQC, HQC; evaluated over three consecutive days).

Matrix	Analyte Compounds	Nominal Conc (μg·mL^−1^)	Intra-Day			Inter-Day		
			Measured Conc (μg·mL^−1^)	RSD (%)	RE (%)	Measured Conc (μg·mL^−1^)	RSD (%)	RE (%)
Plasma	Punicalagin	1	1.07 ± 0.06	6.02	7.21	1.08 ± 0.01	1.04	7.73
		3	3.35 ± 0.13	3.93	11.59	3.02 ± 0.10	5.05	0.6
		12.5	12.55 ± 1.52	12.13	0.43	12.24 ± 0.25	2.01	−2.07
		200	203.03 ± 24.53	12.08	1.52	200.44 ± 3.44	1.71	0.22
Urine	Urolithin A	0.1	0.10 ± 0.01	14.76	0.97	0.10 ± 0.003	3.24	4.65
		5	5.08 ± 0.34	6.66	1.63	5.08 ± 0.09	1.83	1.58
		25	22.32 ± 2.38	10.65	−10.71	23.51 ± 1.09	4.63	−5.98
Feces	Punicalagin	2	2.22 ± 0.13	6.01	10.99	2.22 ± 0.03	1.21	10.79
		20	22.21 ± 0.98	4.42	11.07	21.77 ± 0.63	2.91	3.53
		160	162.14 ± 5.27	3.25	1.34	157.56 ± 4.59	2.91	−1.22
	Punicalin	0.375	0.375 ± 0.017	4.45	0.13	0.388 ± 0.012	2.96	3.51
		3.75	3.36 ± 0.25	7.5	−10.51	3.44 ± 0.21	6.11	−8.31
		30	29.40 ± 2.04	6.92	−1.99	28.45 ± 1.19	4.2	−5.17
	Ellagic acid	0.2	0.21 ± 0.03	13.14	6.08	0.21 ± 0.01	3.63	4.19
		2	1.92 ± 0.16	8.33	−3.77	1.99 ± 0.06	2.98	−0.57
		16	15.70 ± 1.31	8.35	−1.86	15.34 ± 0.70	4.58	−4.13
	Urolithin C	0.0625	0.0605 ± 0.0066	10.98	−3.2	0.0599 ± 0.0037	6.19	−4.1
		0.625	0.615 ± 0.030	4.85	−1.62	0.623 ± 0.007	1.18	−0.31
		5	5.04 ± 0.37	7.25	0.82	4.97 ± 0.07	1.38	−0.58
	Urolithin A	0.125	0.115 ± 0.010	8.88	−7.81	0.128 ± 0.014	10.57	2.37
		1.25	1.16 ± 0.06	4.98	−7.06	1.18 ± 0.04	3.74	−5.5
		10	10.09 ± 1.01	9.98	0.94	10.40 ± 0.34	3.27	3.98

**Table 3:** Extraction recovery and matrix effects for the determination of punicalagin in rat plasma, urolithin A in urine, and punicalagin, punicalin, ellagic acid, urolithin C, and urolithin A in feces using the UPLC-MS/MS method (*n* = 6; three concentration levels: low, medium, high).

Matrix	Analyte Compounds	Nominal Conc (μg·mL^−1^)	Extract Recovery		Matrix Effect	
			Mean ± SD(%)	RSD (%)	Mean ± SD(%)	RSD (%)
Plasma	Punicalagin	3	94.58 ± 11.83	12.51	85.49 ± 3.57	4.18
		12.5	87.27 ± 11.79	13.51	101.57 ± 11.19	11.02
		200	92.43 ± 5.54	5.99	113.64 ± 12.73	11.2
Urine	Urolithin A	0.1	105.28 ± 10.23	9.71	101.93 ± 12.86	12.62
		5	103.17 ± 8.09	7.84	94.83 ± 9.55	10.07
		25	111.04 ± 6.70	6.03	105.58 ± 10.54	9.99
Feces	Punicalagin	2	94.01 ± 8.72	9.27	95.90 ± 9.00	9.39
		20	96.53 ± 3.61	3.74	85.87 ± 4.23	4.92
		160	91.29 ± 6.33	6.93	96.65 ± 2.11	2.18
	Punicalin	0.375	108.44 ± 5.76	5.31	89.66 ± 11.72	13.07
		3.75	101.24 ± 10.39	10.26	99.49 ± 14.08	14.15
		30	98.50 ± 9.57	9.71	110.69 ± 6.60	5.96
	Ellagic acid	0.2	94.54 ± 13.21	13.97	97.76 ± 13.05	13.35
		2	100.25 ± 11.49	11.46	104.20 ± 6.86	6.59
		16	97.12 ± 7.62	7.85	111.10 ± 8.63	7.77
	Urolithin C	0.0625	102.08 ± 14.34	14.05	98.70 ± 7.90	8
		0.625	106.65 ± 7.44	6.97	98.84 ± 7.75	7.84
		5	99.54 ± 10.82	10.87	113.14 ± 6.44	5.69
	Urolithin A	0.125	108.13 ± 8.90	8.23	95.97 ± 9.68	10.09
		1.25	105.08 ± 7.87	7.49	103.65 ± 13.05	12.59
		10	111.60 ± 15.61	13.99	99.14 ± 7.91	7.98

**Table 4:** Stability under different storage conditions for the determination of punicalagin in rat plasma, urolithin A in urine, and punicalagin, punicalin, ellagic acid, urolithin C, urolithin A in feces using the UPLC-MS/MS method (*n* = 6; three concentration levels: low, medium, high).

Matrix	Storage Conditions	Analyte Compounds	Nominal Conc (μg·mL^−1^)	Measured Conc (μg·mL^−1^)	RSD (%)	RE (%)
Plasma	Short term stability	Punicalagin	3	3.03 ± 0.26	8.66	1.04
			12.5	13.27 ± 0.93	7.00	6.16
			200	197.28 ± 10.29	5.22	−1.36
	Frozen-thaw stability		3	3.20 ± 0.30	9.22	6.79
			12.5	12.62 ± 1.48	11.77	0.94
			200	194.02 ± 16.80	8.66	−2.99
	Long term stability		3	3.12 ± 0.35	11.21	3.94
			12.5	13.43 ± 0.48	3.55	7.48
			200	195.72 ± 23.54	12.03	−2.14
Urine	Short term stability	Urolithin A	0.1	0.10 ± 0.01	13.48	4.00
			5	5.31 ± 0.24	4.55	6.18
			25	21.77 ± 0.95	4.37	−12.92
	Frozen-thaw stability		0.1	0.10 ± 0.01	10.41	3.72
			5	5.13 ± 0.37	7.25	2.63
			25	21.82 ± 0.66	3.02	−12.74
	Long term stability		0.1	0.10 ± 0.01	12.75	0.22
			5	4.89 ± 0.48	9.74	−2.22
			25	24.49 ± 1.45	5.93	−2.05
Feces	Short term stability	Punicalagin	2	2.21 ± 0.15	6.88	10.72
			20	22.26 ± 0.59	2.67	11.31
			160	156.64 ± 3.75	2.39	−2.10
		Punicalin	0.375	0.40 ± 0.03	6.46	6.76
			3.75	3.48 ± 0.34	9.66	−7.16
			30	27.52 ± 1.51	5.50	−8.26
		Ellagic acid	0.2	0.21 ± 0.01	6.66	6.81
			2	2.00 ± 0.09	4.68	−0.10
			16	15.13 ± 1.23	8.15	−5.46
		Urolithin C	0.0625	0.0650 ± 0.0062	9.59	4.04
			0.625	0.653 ± 0.038	5.83	4.47
			5	4.85 ± 0.42	8.58	−2.92
		Urolithin A	0.125	0.128 ± 0.015	11.93	2.20
			1.25	1.21 ± 0.10	8.42	−3.19
			10.00	10.67 ± 0.95	8.89	6.72
	Frozen-thaw stability	Punicalagin	2	2.25 ± 0.19	8.50	12.41
			20	21.87 ± 0.65	2.99	9.33
			160	161.48 ± 8.66	5.36	0.92
		Punicalin	0.375	0.375 ± 0.027	7.13	−0.08
			3.75	3.61 ± 0.40	11.09	−3.70
			30	27.47 ± 1.75	6.37	−8.42
		Ellagic acid	0.2	0.20 ± 0.02	10.72	1.55
			2	1.90 ± 0.21	11.03	−4.75
			16	15.27 ± 0.74	4.85	−4.59
		Urolithin C	0.0625	0.0657 ± 0.0078	11.90	5.06
			0.625	0.649 ± 0.073	11.30	3.78
			5	4.84 ± 0.20	4.13	−3.14
		Urolithin A	0.125	0.129 ± 0.016	12.44	3.39
			1.25	1.14 ± 0.13	11.01	−9.17
			10.00	10.69 ± 0.45	4.20	6.89
	Long term stability	Punicalagin	2	2.14 ± 0.18	8.22	6.87
			20	20.25 ± 1.85	9.16	1.27
			160	154.89 ± 6.95	4.49	−3.19
		Punicalin	0.375	0.395 ± 0.030	7.65	5.41
			3.75	4.25 ± 0.49	11.46	13.43
			30	25.81 ± 1.33	5.17	−13.97
		Ellagic acid	0.2	0.17 ± 0.02	10.86	−12.69
			2	1.95 ± 0.12	5.97	−2.28
			16	14.94 ± 1.17	7.86	−6.61
		Urolithin C	0.0625	0.0618 ± 0.0049	7.91	−1.06
			0.625	0.685 ± 0.057	8.31	9.65
			5	4.58 ± 0.36	7.83	−8.33
		Urolithin A	0.125	0.120 ± 0.006	4.64	−4.21
			1.25	1.17 ± 0.05	4.53	−6.11
			10.00	10.25 ± 0.89	8.68	2.54

**Table 5:** Noncompartmental pharmacokinetic parameters of Punicalagin in systemic circulation following intravenous and intraportal administration at 10 mg·kg^−1^ to rats (Mean ± SD, *n* = 6).

Parameters	Punicalagin	
	Intravenous Administration	Intraportal Administration
T_1/2_ (h)	19.14 ± 10.75	18.75 ± 6.22
C_max_ (μg·mL^−1^)	56.72 ± 15.04	35.97 ± 10.37
AUC_0–24h_ (μg·h·mL^−1^)	250.29 ± 50.33	215.41 ± 49.98
T_max_ (h)	0.083 ± 0.00	0.083 ± 0.00
V_d_ (L/kg)	0.61 ± 0.18	0.78 ± 0.27
CL (L/h/kg)	0.025 ± 0.007	0.029 ± 0.004
MRT_0–∞_ (h)	27.76 ± 13.66	25.95 ± 7.10
F_h_%	—	86.06%
ER_h_%	13.94%	

The AUC_0–24h_ values between the portal vein administration group and the intravenous administration group were compared using a *t*-test. A *p* value < 0.05 * indicates a statistically significant difference.

**Table 6:** Noncompartmental pharmacokinetic parameters of Punicalagin. Parameters in the systemic circulation after oral administration at 200 mg·kg^−1^, and parameters in the systemic and portal circulations after intraduodenal administration at 200 mg·kg^−1^ in rats (Mean ± SD, *n* = 6).

Parameters	Punicalagin-id		Punicalagin-ig
	Systemic Circulation	Portal Vein	Systemic Circulation
T_1/2_ (h)	21.21 ± 8.57	16.90 ± 5.25	22.67 ± 5.41
C_max_ (μg·mL^−1^)	13.28 ± 5.47	13.99 ± 2.42	12.35 ± 4.06
AUC_0–24h_ (μg·h·mL^−1^)	170.95 ± 84.08	175.78 ± 13.79	159.85 ± 45.66
T_max_ (h)	2.00 ± 1.10	1.83 ± 0.37	2.17 ± 0.90
V_d_ (L/kg)	22.90 ± 12.49	16.67 ± 3.15	21.90 ± 7.56
CL (L/h/kg)	0.71 ± 0.15	0.73 ± 0.15	0.69 ± 0.20
MRT_0–∞_ (h)	31.05 ± 11.43	24.59 ± 7.82	33.11 ± 7.31
F_a_·F_g_%	—	4.05%	—
ER_g_%	95.95%		
F_G_%	93.51%		
F%	—	—	3.19%
F_meas_%	3.49%		

Pairwise comparisons of the AUC_0–24h_ values among the systemic circulation group, portal circulation group after intraduodenal administration, and the oral administration group were performed using one-way ANOVA. A *p* value < 0.05 * indicates a statistically significant difference.

**Table 7:** Quality control (QC) sample concentrations of the analytes in three matrices.

Matrix	Analyte Compounds	Low (μg·mL^−1^)	Middle (μg·mL^−1^)	High (μg·mL^−1^)
Plasma	Punicalagin	3.0	12.5	200
Feces	Punicalagin	2.0	20	160
	Punicalin	0.375	3.75	30
	Ellagic acid	0.20	2	16
	Urolithin C	0.0625	0.625	5.0
	Urolithin A	0.125	1.25	10
Urine	Urolithin A	0.10	5	25

**Table 8:** Multiple reaction monitoring (MRM) parameters for punicalagin, punicalin, ellagic acid, urolithin C, urolithin A, and the internal standard (IS) in the negative ion mode.

Analyte Compounds	Retention Time/min	Precursor Ion (*m*/*z*)	Fragment/V	Production (*m*/*z*) Quantitative/Qualitative	Collision Energy/V
Punicalagin	3.07/3.31	541	80	301/275	76
Punicalin	2.86	781	42	299/601	40
Ellagic acid	5.21	301	24	229/257	20
Urolithin C	5.12	243	30	187/198	30
Urolithin A	5.44	227	45	182/198	35
Paeoniflorin	4.45	525	40	121/165	20

## Data Availability

The data presented in this study are available upon request from the corresponding author.

## Abbreviations

The following abbreviations are used in this manuscript:

ER_g_	intestinal extraction ratio
ER_h_	hepatic extraction ratio
F_a_	fraction absorbed
F_g_	fraction that avoids intestinal metabolism
F_G_	gastric bioavailability
F_h_	hepatic bioavailability
F_meas_	oral bioavailability
AUC_id_	AUC after intraduodenal administration
AUC_ig_	AUC after intragastric administration
AUC_ipv_	AUC after intraportal (portal vein) administration
AUC_iv_	AUC after intravenous administration
C_max_	Maximum concentration
CL	clearance
T_max_	time to reach Cmax
V_d_	volume of distribution
MRT	Mean Residence Time
R_b_	blood-to-plasma ratio
Q_por_	portal blood flow rate
UPLC-MS/MS	Ultra-Performance Liquid Chromatography–Tandem Mass Spectrometry
IS	Internal standard
MRM	Multiple reaction monitoring
RE	Relative error
RSD	Relative standard deviation
LLOQ	Lower limit of quantification
QC	Quality control
LQC	Low Quality Control
MQC	Mid Quality Control
HQC	High Quality Control
